# The Apparent pH Stability of Antibiotic Preparations Used for Uterine Infusions in Mares

**DOI:** 10.3390/ani16030382

**Published:** 2026-01-26

**Authors:** Mariana Polesso Mazzuchini, Igor F. Canisso

**Affiliations:** 1 Department of Veterinary Clinical Medicine, College of Veterinary Medicine, University of Illinois Urbana-Champaign, Urbana, IL 61802, USA; mp.mazzuchini@unesp.br; 2 School of Veterinary Medicine and Animal Science, São Paulo State University, Botucatu 18618-970, São Paulo, Brazil

**Keywords:** antibiotic, biofilm, endometritis, mare, uterine infusion

## Abstract

Infectious endometritis is a primary cause of subfertility in mares. Many manuals and guidelines are available for administering intrauterine infusions of antibiotics and biofilm-disrupting solutions, detailing concentrations, vehicle solutions, and buffers. However, the stability of these combinations has not been documented. Therefore, our study investigated how factors such as storage temperature, concentration, buffer types, combinations of biofilm disruptors, and vehicle solutions affected pH stability over 24 h in uterine infusion preparations commonly used by veterinary theriogenologists to treat endometritis in mares. In experiment 1, amikacin, ampicillin, ceftiofur, ciprofloxacin, gentamicin, penicillin G potassium, penicillin G procaine, and ticarcillin clavulanate were diluted in saline and lactated Ringer’ s solution and stored at 5 ° C, 21 ° C, and 37 ° C for 24 h. Solutions were evaluated for pH and physical characteristics at 0, 1, 3, 6, and 24 h after storage. In experiment 2, 1- and 2-g doses of amikacin, ampicillin, and gentamicin were compared, evaluating the same volume at different concentrations and their stability over 24 h. Experiment 3 combined biofilm chelators (i.e., Tris-EDTA, hydrogen peroxide, and dimethyl sulfoxide) with antibiotic solutions and evaluated interactions among products. Experiment 4 compared the stability of each antibiotic diluted in saline and lactated Ringer’ s solution. Statistical analysis was performed using GraphPad Prism 9.3.0. Significance was set at *p* < 0.05. The results indicated that the stability of antibiotic solutions for uterine infusions in mares is significantly influenced by storage conditions and pH fluctuations. Solutions containing aminoglycosides showed an increase in pH over time, suggesting that buffering agents like sodium bicarbonate can enhance stability. In contrast, other antibiotics exhibited a decrease in pH, particularly at elevated temperatures, which may reduce their effectiveness. In conclusion, the pH stability of uterine infusions is affected by various storage conditions and vehicles, underscoring the importance of evaluating antibiotic treatments for quality control. While pH changes were observed, the potential impact on the overall stability or antimicrobial activity of the solutions requires further investigation.

## Simple Summary

Endometritis is the primary cause of subfertility in mares. Treatment often involves ecbolics, antibiotics, and uterine lavage. Antibiotics may be administered systemically or directly infused into the uterus. Although many recommendations exist on preparing uterine infusions in mares, their effectiveness has not been thoroughly evaluated. This study aimed to assess in vitro the apparent stability of various preparation methods for antibiotic uterine infusions based on these recommendations. The most frequently used antibiotics in clinical practice were tested, and guidelines for field veterinarians were developed.

## 1. Introduction

Antibiotics are critical for treating bacterial endometritis in mares [1,2]. They are typically administered as uterine infusions or systemically, and less commonly both. The route of administration varies with clinician preferences and experience. Some favor uterine infusion of antibiotics, noting that it delivers the drug directly into the uterine lumen (i.e., targeting the site of infection), achieves high local concentrations, minimizes systemic side effects, and appears to have higher efficacy in clinical practice. Conversely, uterine infusion of antibiotics is thought to disrupt the reproductive tract’s uterine microbiome, favoring the development of fungal overgrowth and mycotic endometritis [3,4].

Using antibiotics systemically has the advantage of being easier to administer. Many times, farm staff, technicians, or owners can systemically administer antibiotics without needing a veterinarian for it; conversely, uterine infusions require a veterinarian to prepare and execute the procedure. It is also believed that the systemic administration of antibiotics can penetrate deeper layers of the uterus and cause less irritation to the endometrium. However, it is associated with adverse effects such as colitis, diarrhea, and systemic reactions, and may potentially contribute to the development of antibiotic resistance, as the antibiotics disseminate throughout the body, including the gastrointestinal tract, where a substantial microbiome exists and may be negatively impacted by systemic antimicrobial agents [5]. However, some antibiotics can be cost-prohibitive for use systemically in adult horses.

Classes of antibiotics commonly used as uterine infusions include aminoglycosides (e.g., amikacin and gentamicin), cephalosporins (e.g., ceftiofur), and beta-lactams (e.g., ticarcillin, penicillin, extended-spectrum penicillin, imipenem, and ampicillin) [1,2]. Antibiotics ought to be chosen on the basis of bacterial culture results, microorganism identification, and susceptibility testing to prevent the emergence of antibiotic resistance This approach ensures the efficacy of uterine infusions and promotes the even distribution of the solution within the uterus [1,6,7]. Additionally, solutions capable of disrupting biofilms are required to degrade the extracellular polymeric substances that protect microbial cells and inhibit antibiotic contact, consequently reducing antimicrobial resistance [8,9].

Several recommendations exist for the use of uterine infusions of antibiotics and antibiofilm disruption agents in mares, yet they have not been critically evaluated through in vitro or in vivo studies [7,8,10,11,12,13,14,15,16]. Different protocols employing the same drugs have been documented, varying in concentrations, solutions, buffers, and vehicles. To our knowledge, no research has examined the stability of these uterine preparations under such diverse conditions. Moreover, factors like storage temperature changes and minor pH variations have been reported to affect the antimicrobial efficacy of antibiotics [17].

In clinical practice, veterinarians often prepare antibiotic solutions and leave them at various temperatures for times up to 24 h. Therefore, this study aimed to assess the guidelines for preparing uterine infusions in mares; specifically, we determined the impact of storage temperature, time of storage, type of diluent, concentration of antibiotics, and interactions with biofilm disruptors on pH and apparent solution stability.

## 2. Materials and Methods

### 2.1. Study Design

The study assessed the potential stability of antibiotic preparations for use as uterine infusions in mares. The pH, turbidity, and sedimentation were used to determine apparent solution stability over time. These evaluations were conducted immediately after preparation and under different storage temperatures to mimic potential field conditions, since often, antibiotics are prepared and sometimes not used immediately by practitioners. As antibiotics are often prepared in various concentrations, the effect of concentration was assessed in experiment 2. Chemicals denominated biofilm disruptors that are commonly used in combination with antibiotics; thus, the stability and physical properties of antibiotic and biofilm disruptor solutions commonly used for uterine infusion in mares were assessed in experiment 3. Equine practitioners have saline solution (NaCl 0.9%) and lactated Ringer’s solution (LRS) available at their disposal, and many question whether one solution is better than the other for uterine infusions of antibiotics; thus, these two solutions were assessed in experiment 4. Stock solutions and antibiotics were maintained in a temperature-controlled environment at 21 °C and protected from light to preserve stability.

#### 2.1.1. Experiment 1: Influence of Storage Temperatures on Antibiotic Solutions’ Stability

Experiment 1 investigated the apparent pH stability of antibiotic solutions that are used for uterine infusions in mares over 24 h, stored in a refrigerator set at 5 °C, over a countertop at room temperature, and in an incubator at 37 °C. Various antibiotics were evaluated in triplicate at multiple timepoints immediately after preparation (time 0) and at 1, 3, 6, and 24 h after storage. These timelines were chosen in an attempt to mimic field conditions, where the uterine solution may be prepared in advance, and practitioners are uncertain what temperature to store it at and for how long it can be stored.

#### 2.1.2. Experiment 2: Influence of Antibiotic Concentration on Solutions’ Stability

Experiment 2 compared two concentrations of antimicrobials commonly prescribed in clinical practice. Amikacin, ampicillin, and gentamicin uterine infusions containing 1 and 2 g in 60 mL solutions were stored and evaluated under the same conditions as described in experiment 1, and the stability of the antibiotics was compared between the two concentrations.

#### 2.1.3. Experiment 3: Stability of Non-Antibiotic Biofilm Disruptors and Antibiotic-Associated Solutions

Experiment 3 evaluated the stability of the association between non-antibiotic and antibiotic solutions used for biofilm disruption in mares’ uteruses. Dimethyl sulfoxide, hydrogen peroxide, and Tris-EDTA were the non-antibiotic compounds selected after previous recommendations [8,10,16], while amikacin, ceftiofur, and ciprofloxacin were the antibiotics [10]. The endpoints for stability measured in this experiment were similar to those of experiment 1.

#### 2.1.4. Experiment 4: Influence of Vehicle on Solutions’ Stability

Experiment 4 was performed to compare saline solution (NaCl 0.9%) and lactated Ringer’s solution were evaluated as diluents for the different uterine preparations. Universally, those two solutions are the most widely used.

### 2.2. Solutions

#### 2.2.1. Antibiotics

The evaluation of the most common antibiotics recommended for uterine infusions in mares involved assessing a final infusion volume of 60 mL. This volume was selected to replicate the commonly recommended uterine infusion volume in mares. Solutions were prepared, adhering to specified concentrations, and the required volume was measured using a calibrated pipette and a scale for accurate weight measurement. Aminoglycoside solutions (i.e., amikacin and gentamicin) for uterine infusions were buffered using 10 mL of 8.4% sodium bicarbonate (NaHCO_3_), achieving a final volume of 60 mL (Table 1). Cephalosporin and fluoroquinolone solutions (i.e., ceftiofur and ciprofloxacin) for uterine infusion in mares were described in Table 2. Penicillin-based uterine infusions (i.e., ampicillin, penicillin G potassium, penicillin G procaine, and ticarcillin clavulanate) are described in Table 3. The manufacturers from whom they were purchased are listed in Table S1. Two vehicles were used to dilute the antibiotic solutions: saline solution (NaCl 0.9%) and lactated Ringer’s solution.

#### 2.2.2. Biofilm Disruptors and Antibiotic Association

Combinations of antibiotics and non-antibiotic solutions have been described to disrupt biofilm and improve antimicrobial effectiveness in mares with infectious endometritis [8]. The solutions were diluted, achieving a final volume of 60 mL using NaCl 0.9% and lactated Ringer’s solution as the vehicle, as previously described for the antibiotic infusions. The concentration and volume of the antibiotics were the same as previously described for AMK, CEFT, and CIPRO in Table 1 and Table 2, and their dilutions in non-antibiotic compounds are described in Table 4. AMK-based solutions were buffered using 10 mL of NaHCO_3_ 8.4%.

### 2.3. Storage

Following thorough homogenization, the prepared solutions were meticulously aliquoted into three individual 15 mL conical tubes to achieve consistency across samples. Each tube was designated to a unique temperature regimen: one stored at a refrigeration temperature of 5 °C, another at ambient room temperature (21 °C), and the third incubated at 37 °C to simulate physiological conditions. The pH was measured immediately after preparation (time 0) and subsequently at 1, 3, 6, and 24 h after storage.

### 2.4. pH Stability

The pH was measured in triplicate using a handheld pocket meter (LAQUAtwin pH-22, HORIBA Advanced Techno Co., Irvine, CA, USA) composed of a flat sensor (pH Sensor S010, HORIBA Advanced Techno Co.) that is known to present a high accuracy (±0.01) and up to 3 calibration points. The calibrations were performed immediately before the first evaluation of each solution and after every three samples using three standard pH solutions (VWR International LLC, Radnor, PA, USA): pH 4 ± 0.01 (Cat. #BDH5024), pH 7± 0.01 (Cat. #BDH5052), and pH 10 ± 0.01 (Cat. #BDH5072).

### 2.5. Statistical Analysis

Statistical analysis was performed using GraphPad Prism 9.3.0. To assess the Gaussian distribution of the data, a Shapiro–Wilk normality test was conducted, ensuring a suitable parametric statistical analysis. In experiments 1 and 3, to compare the influence of storage conditions on antibiotic solutions, data were organized in groups according to the temperature of storage conditions (i.e., 5 °C, 21 °C, and 37 °C), where each row represented different timepoints (i.e., time 0, 1, 3, 6, and 24 h). Then, two separate two-way ANOVAs were performed: one to evaluate the effect within rows in the same storage temperature group and another assessing the simple effect within columns across the different timepoints.

In experiment 2, to measure the influence of the drug’s concentration on the stability of the antibiotic solution, a multiple unpaired test with Welch’s correction was performed to compare the pH from each antibiotic solution at the same time point across the different concentrations. In experiment 4, data were reorganized into one table for each storage temperature, divided into two groups based on the vehicle used (saline or lactated Ringer’s solution). Each row represented different timepoints, and matched values were stacked into sub-columns. A two-way ANOVA was performed, comparing the means of each cell to evaluate the differences between saline and lactated Ringer’s vehicle-based solutions. Significance was set at *p* < 0.05 in all analyses.

## 3. Results

### 3.1. Experiment 1: Influence of Storage Conditions on Antibiotic Solutions’ Stability

The different antibiotic preparations for uterine infusions showed remarkable variations in pH from initial dilution to final storage in different temperatures (Table S2). Amikacin (AMK), both diluted in saline solution and lactated Ringer’s solution, increased the pH over time for all temperatures (*p* < 0.05). Additionally, temperature-dependent differences across the timepoints were observed during the initial 0 to 3 h after storage, and the pH was stabilized after 6 h for all groups (*p* < 0.05). Conversely, ampicillin (AMP) solutions decreased the pH across the different timepoints for all temperatures; however, higher storage temperatures, especially at 37 °C, reduced the pH over 24 h for all ampicillin solutions (*p* < 0.05).

Ceftiofur sodium (CEFT) solutions, both diluted in saline and lactated Ringer’s solutions, stored at 5 °C, maintained the pH over the primary 6 h; after 24 h (*p* > 0.05), CEFT + S increased the pH when compared to hour 0 (*p* < 0.05). High temperatures decreased the pH over 24 h, with a low pH at 37 °C storage, reduced when compared to 5 °C and 21 °C (*p* < 0.05); besides that, slight color changes were observed in the samples stored overnight at 37 °C. Ciprofloxacin (CIPRO) solution diluted in lactated Ringer’s solution increased the pH at 5 °C over 24 h, while when stored at warm temperatures, no changes were observed. However, ciprofloxacin diluted in saline solution decreased the pH for all temperatures and sedimented over 24 h (*p* < 0.05).

Gentamicin (GEN) solutions, whether diluted with lactated Ringer’s solution or saline, showed a gradual increase in pH over time. Notably, storing them at 37 °C had the most significant impact on pH stability during the 24 h period. Generally, pH levels stabilized by around 6 h and remained steady by the 24 h across different temperatures. Conversely, solutions containing penicillin G potassium (KPEN) and penicillin G procaine (PPG) displayed minimal pH fluctuations when stored at 5 °C, indicating stable conditions. However, at higher temperatures, especially 37 °C, there was a notable decline in pH over time. Specifically, penicillin G potassium stored at 37 °C for 24 h experienced acidity (*p* < 0.05), suggesting faster degradation or chemical reactions.

Ticarcillin clavulanate (TIC-CLAV) solutions displayed notable changes in pH depending on how they were stored. Solutions kept at room temperature (21 °C) remained stable throughout the 24 h period. Refrigerated solutions increased the pH, whereas solutions stored at higher temperatures (5 °C and 37 °C) tended to be more acidic after 3 h. Color changes were observed after 24 h in the solutions that were kept in warmer conditions when compared to time 0.

**Figure 1:**
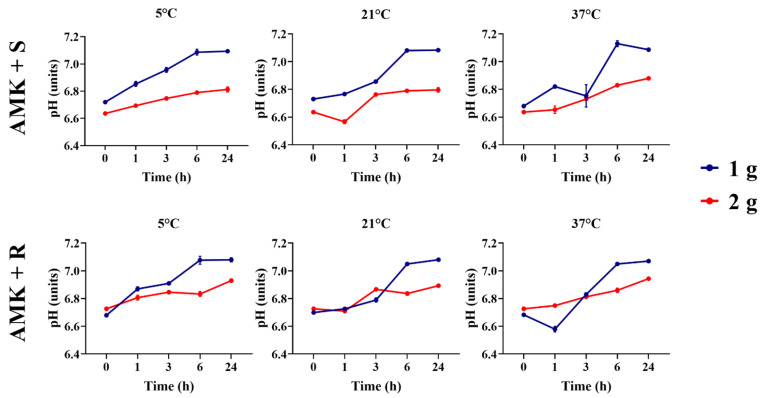
Comparison of amikacin solutions’ pHs between 1 g and 2 g concentrations across timepoints and temperatures. AMK + S, amikacin diluted in saline solution. AMK + R, amikacin diluted in lactated Ringer’s solution.

Amikacin diluted in both saline solution and lactated Ringer’s solution, whether containing 1 or 2 g, generally showed an increased pH over 24 h for most temperatures and timepoints. Additionally, amikacin at 1 and 2 g diluted in lactated Ringer’s solution did not show any difference between pH over the first three hours when stored at 21 °C and over the first hour when stored at 5 °C (*p* > 0.05). Amikacin stored at 37 °C showed a difference in all timepoints when comparing the pHs of the solutions containing 1 and 2 g (*p* > 0.05, Figure 1).

**Figure 2:**
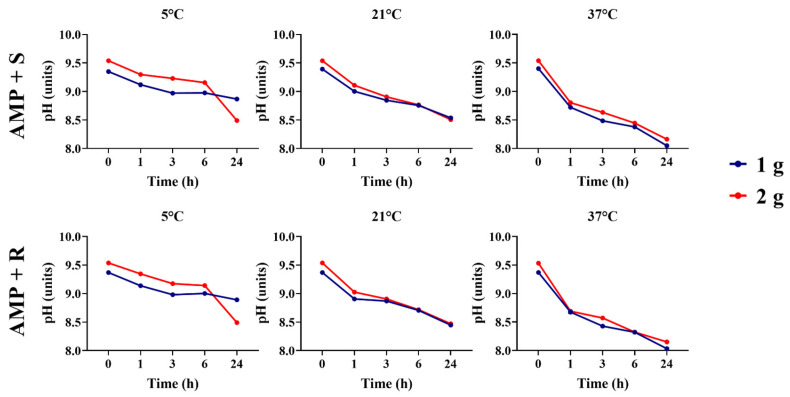
Comparison of ampicillin solutions’ pHs between 1 g and 2 g concentrations across timepoints and temperatures. AMP + S, ampicillin diluted in saline solution. AMP + R, ampicillin diluted in lactated Ringer’s solution.

Ampicillin solutions containing 1 g and 2 g, both diluted in saline and lactated Ringer’s solution, exhibited pH differences over 24 h when stored at 5 °C. A higher concentration of the alkaline antibiotic increased the pH compared to the two concentration solutions (*p* < 0.05). Across all temperatures, both solutions exhibited a decrease in pH over time (Figure 2).

**Figure 3:**
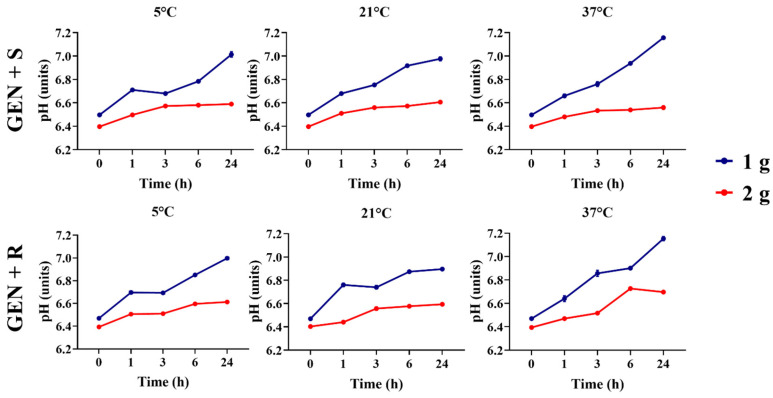
Comparison of gentamicin solutions’ pHs between 1 g and 2 g concentrations across timepoints and temperatures. GEN + S, gentamicin diluted in saline solutions. GEN+ R, gentamicin diluted in lactated Ringer’s solution.

Gentamicin 1 and 2 g showed differences between all temperatures and timepoints (*p* < 0.05, Figure 3). Both antibiotic concentrations showed an increase in pH over time (*p* < 0.05); however, gentamicin at 2 g displayed less variation over 24 h and maintained a lower pH compared to gentamicin at 1 g at all time points (*p* > 0.05).

### 3.2. Experiment 3: Stability of Non-Antibiotic Biofilm Disruptors and Antibiotic-Associated Solutions

Dimethyl sulfoxide, when associated with ceftiofur sodium (DMSO + CEFT) or ciprofloxacin (DMSO + CIPRO), increased the pH for both antibiotic solutions over all timepoints compared with the solutions that were just diluted in saline or lactated Ringer’s solution (Table 1 and Table 5; *p* < 0.05). Ceftiofur–DMSO associations decreased the pH after 3 h storage in higher temperatures (37 °C) while remaining more stable when stored at refrigeration and room temperatures (5 °C and 21 °C, respectively). Ciprofloxacin–DMSO saline-based solutions exhibited stable pH levels, whereas those in lactated Ringer’s solution started at higher initial pH values. Over time, saline solutions showed minor pH fluctuations (*p* < 0.05), whereas solutions in lactated Ringer’s solution demonstrated greater stability, particularly after 24 h at higher temperatures (Table 5).

The addition of hydrogen peroxide to amikacin antibiotic solution (H_2_O_2_ + AMK), both diluted in saline solution or lactated Ringer’s solution, increased the pH over time in all storage conditions (Table 6; *p* < 0.05). A refrigeration temperature (5 °C) increased the pH of both amikacin + hydrogen peroxide solutions significantly after 1 h storage, being stable up to 6 h; after that, a increase was observed at the 24 h evaluation. Room temperature (21 °C) showed a similar increase in pH over 24 h as observed in the other storage temperatures (*p* < 0.05), despite the fact that the variability in amikacin + hydrogen peroxide + saline was lighter than observed at 5 °C and 37 °C up to 6 h. Additionally, the storage of amikacin + hydrogen peroxide solutions at 37 °C increased the pH over 24 h compared to other storage conditions (*p* < 0.05).

Variations in pH were observed in ciprofloxacin + hydrogen peroxide (H_2_O_2_ + CIPRO) solutions over a 24 h period, depending on the storage temperature (Table 6; *p* < 0.05). Temperatures of 21 °C and 37 °C exhibited less pH variation over 6 h compared to refrigerated conditions. Refrigerated storage increased pH levels over 24 h, whereas higher temperatures resulted in a decreased pH over time, particularly in ciprofloxacin + hydrogen peroxide + saline solutions (*p* < 0.05).

The association of Tris-EDTA with antibiotic solutions showed different pH changes over time and under different storage conditions, depending on the antibiotic used (Table 7). Tris-EDTA + AMK solutions, whether diluted in saline or lactated Ringer’s solution, showed a pH increases over time at all storage temperatures after 24 h compared to initial pH levels, with the most pronounced changes observed in solutions stored at 37 °C (Table 7; *p* < 0.05). Conversely, the association of Tris-EDTA + CEFT and Tris-EDTA + CIPRO decreased the pHs of the solutions that were stored at warmer temperatures (i.e., 21 °C and 37 °C), with a decrease observed at 37 °C (*p* < 0.05). Upon refrigeration (5 °C), Tris-EDTA–ceftiofur solutions remained relatively stable, with minimal pH changes over time, while Tris-EDTA–ciprofloxacin solutions slightly reduced the pH up to 6 h storage and then had a minimal increase after 24 h (Table 7, *p* < 0.05).

### 3.3. Experiment 4: Influence of Vehicle on Solutions’ Stability

**Figure 4:**
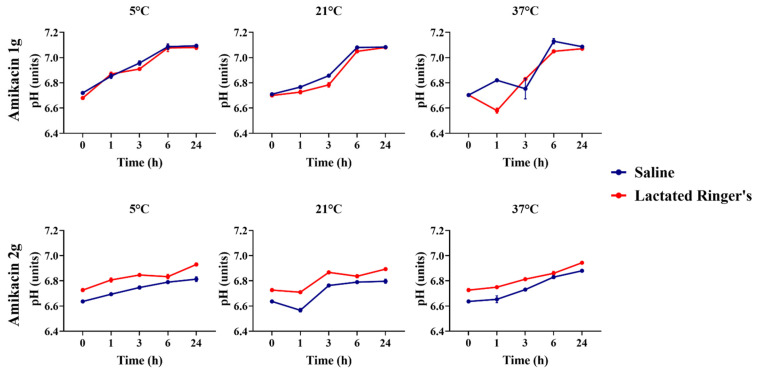
pH comparison of amikacin at 1 g and 2 g diluted in saline or lactated Ringer’s solutions over 24 h.

Amikacin solutions containing 1 g and 2 g, both diluted in saline or lactated Ringer’s solutions, were compared in terms of their pH over time at each temperature that was submitted and according to the solutions that were used as a vehicle (Figure 4). Amikacin at 1 g showed significant pH variations over solutions diluted in saline and lactated Ringer’s solution stored at 21 °C over hour zero (*p* < 0.001), despite that no differences were observed at room temperature solutions in the other timepoint evaluations (*p* > 0.05). 

**Figure 5:**
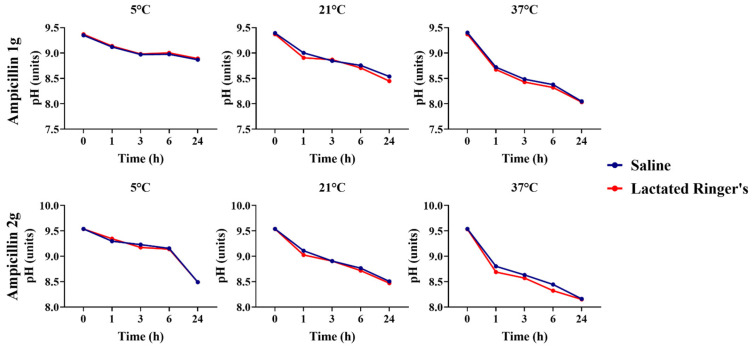
pH comparison of ampicillin at 1 g and 2 g diluted in saline or lactated Ringer’s solutions over 24 h.

Ampicillin at 1 g and 2 g diluted in saline or lactated Ringer’s solutions had their pH measured over 24 h according to the temperature at which they were stored (Figure 5). Amikacin at 1 g varies the pH when stored at 21 °C when comparing saline and lactated Ringer’s solutions as the vehicle at 1 and 24 h measurements (*p* < 0.05); no differences were observed between the ampicillin 1 g pH when comparing the vehicles stored at 5 °C and 37 °C (*p* > 0.05). 

**Figure 6:**
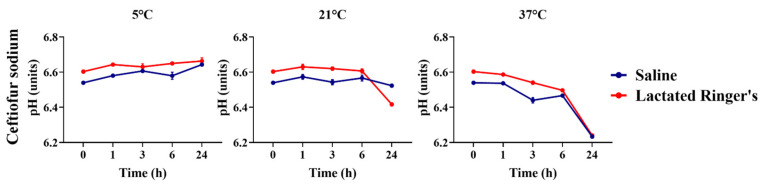
pH comparison of ceftiofur sodium diluted in saline or lactated Ringer’s solutions over 24 h.

Ceftiofur sodium diluted in saline or lactated Ringer’s solution had its pH compared to the vehicle that was used over time and over different storage temperatures (Figure 6). A higher pH was observed for ceftiofur sodium diluted in lactated Ringer’s solution compared to saline solution in almost all timepoints and storage temperatures (*p* < 0.05). 

**Figure 7:**
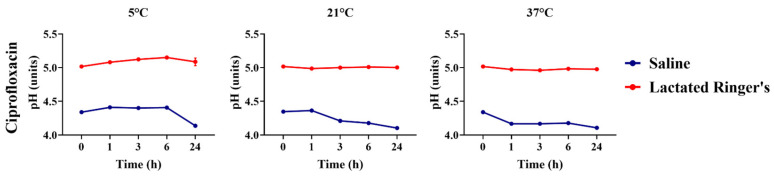
pH comparison of ciprofloxacin diluted in saline or lactated Ringer’s solutions over 24 h.

The pH of ciprofloxacin, diluted in saline or lactated Ringer’s solution, was compared over time and across different storage temperatures, based on the type of vehicle used (Figure 7). Ciprofloxacin diluted in lactated Ringer’s solution showed a higher pH over all storage temperatures and timepoints compared to ciprofloxacin diluted in saline solution (*p* < 0.05).

**Figure 8:**
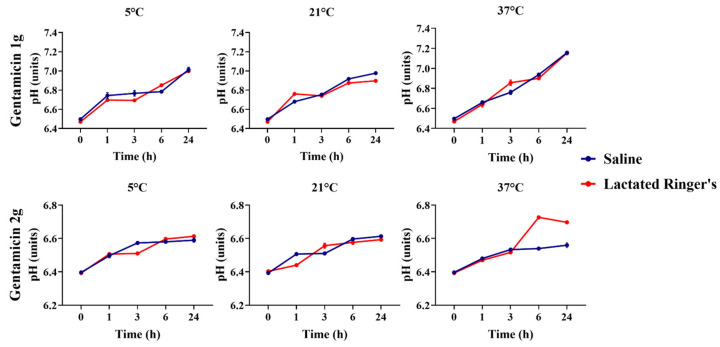
pH comparison of gentamicin at 1 g and 2 g diluted in saline or lactated Ringer’s solutions over 24 h.

The pH of gentamicin at 1 g and 2 g diluted in saline or lactated Ringer’s solutions was measured over 24 h, based on the storage temperature (Figure 8). An increase in pH was observed over time for both gentamicin at 1 g and 2 g in saline and lactated Ringer’s solutions. 

**Figure 9:**
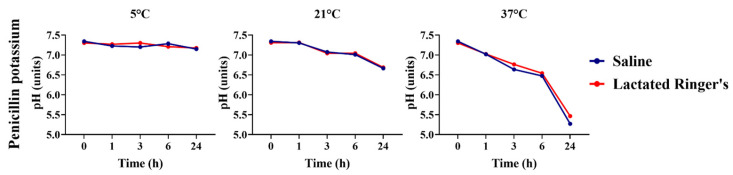
pH comparison of penicillin potassium diluted in saline or lactated Ringer’s solutions over 24 h.

The pH of penicillin G potassium, diluted in either saline or lactated Ringer’s solution, was assessed over time and at various storage temperatures, depending on the type of vehicle (Figure 9). Differences in the pH of the antibiotic diluted in the two vehicles were observed in solutions stored at 5 °C and evaluated after 3 h storage (*p* < 0.05). However, at 6 and 24 h storage, no pH variations were observed between saline or lactated Ringer’s penicillin G potassium diluted solutions conserved under the same conditions (*p* > 0.05). A warmer temperature (37 °C) decreased the pHs of both vehicle-based solutions over time; however, a decrease was observed at 3 and 6 h storage for penicillin G potassium diluted in saline solutions (*p* = 0.01 and *p* = 0.03, respectively).

**Figure 10:**
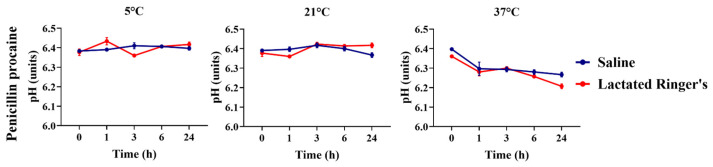
pH comparison of penicillin procaine diluted in saline or lactated Ringer’s solutions over 24 h.

Penicillin G procaine diluted in saline and lactated Ringer’s solutions were compared regarding their pH over 24 h (Figure 10). Solutions stored at 5 °C and 21 °C did not change in terms of pH comparing both vehicles, while 37 °C slightly decreased the pH at 24 h storage evaluation (*p* = 0.04).

**Figure 11:**
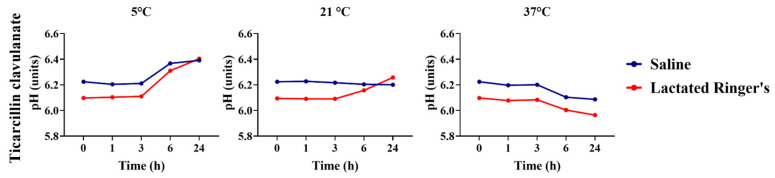
pH comparison of ticarcillin clavulanate diluted in saline or lactated Ringer’s solutions over 24 h.

Ticarcillin clavulanate significantly changed the pH compared to saline or lactated Ringer’s dilution at all storage temperatures up to 6 h evaluations (Figure 11; *p* < 0.05). No changes were observed after 24 h for solutions stored at refrigeration and room temperatures (5 °C and 21 °C, respectively). Changes were observed between the pH of saline and lactated Ringer ’s-based solutions stored at warmer temperatures (37 °C) up to 24 h (*p* < 0.05).

## 4. Discussion

This study aimed to evaluate the variations in antibiotic preparations used for uterine infusions in mares. It also examined combinations of biofilm disruptors alongside antibiotics. A wide range of pH levels was observed among the solutions, with some being acidic and others alkaline. To assess antibiotic stability, solubility,1qA and pH were used as key endpoints. While it is still unclear if storage affects their efficacy in vitro or in vivo, our findings suggest potential guidelines (see Table 5, Table 6 and Table 7) for veterinarians to determine the suitability of these solutions for clinical application [18]. In clinical practice, veterinarians often store solutions at different temperatures. To mirror real-world scenarios, we included common storage conditions found in breeding farm environments, accounting for temperature fluctuations that can happen in the field. This makes our findings more applicable to routine equine practice by highlighting how storage methods may influence the stability of antibiotics used to treat mare endometritis. 

Antibiotics are frequently used to treat endometritis in mares, with selection guided by antimicrobial susceptibility testing. The route of administration is also crucial for improving drug absorption at the infection site and reducing the risk of antimicrobial resistance. Uterine infusion of antibiotics has been a longstanding practice in equine medicine. More recently, the combination of biofilm disruptors with antibiotics has gained popularity and is increasingly used in equine treatment [8,10,16,17,19,20,21]. These combinations have not been extensively studied, but we present some *in vitro* evidence supporting the use of antibiotics and biofilm disruptors in uterine infusions for mares [19]. Since temperature and storage duration influence the stability of these agents, we also provide recommendations for practitioners on storage conditions and temperatures tailored to each uterine preparation. Various antibiotic solutions exhibited different pH changes under different storage conditions. Amikacin and gentamicin, both acidic broad-spectrum aminoglycosides given at intrauterine doses of 1 and 2 g, were consistently buffered to prevent damage to endometrial cells and reduce inflammation [1,22], the pH increased over time, stabilizing after 6 h at all temperatures. This indicates that sodium bicarbonate improves the solution and that its buffering capacity reaches a maximum over time. As a result, acidic solutions could lessen potential damage to epithelial tissue while maintaining buffering capacity for up to 6 h before uterine infusion. In contrast, other antibiotic solutions showed a decrease in pH over time, especially those stored at 37 °C, suggesting that heat exposure might reduce antibiotic effectiveness. 

Amikacin, ampicillin, and gentamicin are frequently administered into mares’ uteruses at doses of 1 or 2 g, depending on the veterinarian’s preference, the mare’s uterine size, and the isolate. When comparing these concentrations for each antibiotic, a higher dose influenced the characteristic pH: acidic drugs lowered the pH at 2 g compared to 1 g, while alkaline drugs raised the pH at higher concentrations. This indicates that adjusting volume could help maintain the original pH or may not be clinically significant; further in vivo studies are needed to confirm this. Additionally, the pH of the solutions remained consistent over time and across different temperatures, suggesting that increasing concentration does not significantly affect their stability.

Ciprofloxacin displayed varying stability profiles under different diluent and temperature conditions. In lactated Ringer’s solution at 5 °C, its pH increased over 24 h, indicating improved stability likely due to favorable thermodynamic interactions. At elevated temperatures, the pH remained stable, suggesting the solution’s equilibrium was preserved. Conversely, when diluted in saline, ciprofloxacin consistently showed a decrease in pH and sedimentation at all temperatures, probably because ionic interactions promote hydrolysis and precipitate formation. These findings emphasize the need to choose suitable diluents and control storage conditions to maintain the stability and effectiveness of ciprofloxacin formulations.

Ticarcillin-clavulanate solutions exhibit significant physicochemical variations depending on storage conditions. Solutions maintained at room temperature (21 °C) demonstrate remarkable pH stability over 24 h, suggesting this temperature range is optimal for preservation. When refrigerated, an increased pH was observed, likely due to shifts in chemical interactions at lower temperatures. Additionally, notable color changes occurred in solutions stored at elevated temperatures after 24 h, suggesting potential oxidative or chemical changes.

The stability of penicillin G potassium and penicillin G procaine solutions was strongly influenced by storage temperature. At 5 °C, both antibiotics maintained stable pH levels, indicating their stability under cooler conditions and minimizing degradation risks. However, increased temperatures, particularly 37 °C, accelerated chemical reactions, leading to significant pH declines and suggesting a higher rate of degradation. This instability at elevated temperatures highlighted the necessity for careful temperature control to preserve the pH of these penicillin formulations, emphasizing the importance of optimized storage conditions for maintaining their therapeutic effectiveness.

Dimethyl sulfoxide, Tris-EDTA, and hydrogen peroxide have been reported to break biofilms and increase antibiotic absorption in mares’ uteruses [7,10,11,23,24]. In addition, these drugs have had their antimicrobial properties proven against many microorganisms. Combinations of these alternative biofilm disruptors and antibiotics have been reported in reproductive clinical practice. Interactions between antibiotics and biofilm disruptors affected the pH of the solutions, suggesting that these associations could impact their efficacy and pH stability. Saline and lactated Ringer’s solution are the primary vehicles recommended for uterine infusions in mares and are also commonly used for uterine lavages [2,12,24]. Although the use of saline or lactated Ringer’s solution affected the pH of some infusions, these solutions generally exhibit consistent variations across the same measurement timepoints and temperatures. Notably, ciprofloxacin infusions showed an important decrease in pH when diluted in saline compared to the other drugs that were assessed, suggesting that lactated Ringer’s vehicle could be advantageous. Variations in pH may indicate chemical degradation of the active pharmaceutical ingredient, while increased turbidity and the presence of sedimentation are indicative of physical instability, potentially resulting from precipitation, crystallization, or aggregation. Additionally, such pH changes have been associated with a decrease in antimicrobial properties in previous studies [17].

## 5. Conclusions

In conclusion, our study highlighted that variations. Notably, solutions containing aminoglycosides demonstrated a consistent increase in pH over time, suggesting that buffering agents like sodium bicarbonate effectively enhance stability. Conversely, other antibiotics showed a decline in pH, particularly at elevated temperatures, which may impair their function. These findings emphasize the importance of optimizing formulation conditions to ensure the efficacy of antibiotic treatments in clinical practice. Further in vitro studies are warranted to evaluate the antimicrobial activity of these infusions under varying storage conditions. Future research should investigate the potential impact of pH variability on antibiotic efficacy. Additionally, employing advanced analytical techniques, such as high-performance liquid chromatography, could yield more detailed information about the degradation pathways of these compounds and allow for correlations with the pH changes observed in this study.

## Figures and Tables

**Table 1:** Aminoglycoside solutions for uterine infusion in mares.

Antibiotic	Name of Solution	Stock Solution/Weighed Portion	Vehicle	Volume
Amikacin (AMK)	AMK-Saline 1 g	4 mL (250 mg/mL)	Saline	46 mL
	AMK-LRS 1 g	4 mL (250 mg/mL)	LRS	46 mL
	AMK-Saline 2 g	8 mL (250 mg/mL	Saline	42 mL
	AMK-LRS 2 g	8 mL (250 mg/mL)	LRS	42 mL
Gentamicin (GEN)	GEN 1 g + Saline	10 mL (100 mg/mL)	Saline	40 mL
	GEN 1 g + LRS	10 mL (100 mg/mL)	LRS	40 mL
	GEN 2 g + Saline	20 mL (100 mg/mL)	Saline	30 mL
	GEN 2 g + LRS	20 mL (100 mg/mL)	LRS	30 mL

Each aminoglycoside solution was buffered with 10 mL of 8.4% NaHCO_3_ to a final volume of 60 mL. Saline, NaCl 0.9%; LRS, lactated Ringer’s solution.

**Table 2:** Cephalosporin and fluoroquinolone solutions for uterine infusion in mares.

Antibiotics	Name of Solution	Stock Solution/Weighed Portion	Vehicle	Volume
Ceftiofur (CEFT)	CEFT-Saline	1 g	Saline	60 mL
	CEFT-LRS	1 g	LRS	60 mL
Ciprofloxacin (CIPRO)	CIPRO-Saline	2 mL (200 mg/mL)	Saline	58 mL
	CIPRO-LRS	2 mL (200 mg/mL)	LRS	58 mL

Saline, NaCl 0.9%; LRS, lactated Ringer’s solution.

**Table 3:** Penicillin solutions for uterine infusion in mares.

Antibiotics	Name of Solution	Stock Solution/Weighed Portion	Vehicle	Volume
Ampicillin (AMP)	AMP 1 g-Saline	1 g	Saline	60 mL
	AMP 1 g-LRS	1 g	LRS	60 mL
	AMP 2 g-Saline	2 g	Saline	60 mL
	AMP 2 g-LRS	2 g	LRS	60 mL
Penicillin G potassium (KPEN)	KPEN-Saline	5,000,000 IU	Saline	60 mL
	KPEN-LRS	5,000,000 IU	LRS	60 mL
Penicillin G procaine (PPG)	PPG-Saline	15 mL (300,000 UI/mL)	Saline	45 mL
	PPG-LRS	15 mL (300,000 UI/mL)	LRS	45 mL
Ticarcillin clavulanate (TIC-CLAV)	TIC-CLAV-Saline	3.1 g	Saline	60 mL
	TIC-CLAV-LRS	3.1 g	LRS	60 mL

Saline, NaCl 0.9%; LRS, lactated Ringer’s solution.

**Table 4:** Non-antibiotic and antibiotic infusions for biofilm disruption in mares with endometritis.

Name of Solution	Non-Antibiotic		Antibiotic		Vehicle Volume
	Compound	Volume	Compound	Dosage	
DMSO + CEFT	DMSO	20 mL	CEFT	1 g	40 mL
DMSO + CIPRO	DMSO	20 mL	CIPRO	400 mg	38 mL
H_2_O_2_ + AMK	H_2_O_2_	20 mL	AMK	1 g	26 mL
H_2_O_2_ + CIPRO	H_2_O_2_	20 mL	CIPRO	400 mg	38 mL
Tris-EDTA + AMK	Tris-EDTA	30 mL	AMK	1 g	16 mL
Tris-EDTA + CEFT	Tris-EDTA	30 mL	CEFT	1 g	30 mL
Tris-EDTA + CIPRO	Tris-EDTA	30 mL	CIPRO	400 mg	28 mL

DMSO, dimethyl sulfoxide; H_2_O_2,_ hydrogen peroxide; Tris-EDTA, Tris base and ethylenediaminetetraacetic acid. Adapted from Ferris [8,10].

**Table 5:** pH (mean ± SEM) stability of dimethyl sulfoxide associated with ceftiofur or ciprofloxacin, both diluted in saline or lactated Ringer’s solution.

	DMSO + CEFT + saline (pH units)		
Time (h)	5 °C	21 °C	37 °C
0	7.7 ± 0 ^A^	7.7 ± 0 ^A^	7.7 ± 0 ^A^
1	7.7 ± 0 ^Ba^	7.8 ± ^Bb^	7.7 ± 0.01 ^Aa^
3	7.6 ± 0.01 ^Ca^	7.8 ± 0 ^ACb^	7.7 ± 0 ^Bc^
6	7.7 ± 0.01 ^Ca^	7.7 ± 0 ^Cb^	7.6 ± 0.01 ^ABa^
24	7.6 ± 0 ^Ca^	7.7 ± 0.01 ^ACa^	6.9 ± 0 ^Cb^
	DMSO + CEFT + lactated Ringer’s solution (pH units)		
Time (h)	5 °C	21 °C	37 °C
0	7.4 ± 0.01 ^A^	7.4 ± 0.01	7.4 ± 0.01 ^A^
1	7.5 ± 0.02 ^Ba^	7.4 ± 0.01 ^b^	7.3 ± 0 ^Bc^
3	7.42 ± 0 ^ABa^	7.4 ± 0.01 ^a^	7.3 ± 0.01 ^Bb^
6	7.4 ± 0.01 ^ABa^	7.4 ± 0.01 ^a^	7.2 ± 0 ^Cb^
24	7.4 ± 0.01 ^ABa^	7.4 ± 0.01 ^a^	6.7 ^Db^
	DMSO + CIPRO + saline (pH units)		
Time (h)	5 °C	21 °C	37 °C
0	4.9 ± 0.01 ^A^	4.9 ± 0.01 ^A^	4.9 ± 0.01 ^AB^
1	4.9 ± 0.02 ^ABab^	4.9 ± 0.01 ^Aa^	4.9 ± 0.01 ^BCb^
3	4.9 ± 0.01 ^Ba^	4.9 ± 0.01 ^Ab^	5.0 ± 0.01 ^CDc^
6	5 ± 0.01 ^Ba^	4.9 ± 0.01 ^ABb^	5.0 ± 0.01 ^Da^
24	5 ± 0.01 ^ABa^	5 ± 0.01 ^Ba^	4.8 ± 0 ^Ab^
	DMSO + CIPRO + lactated Ringer’s solution (pH units)		
Time (h)	5 °C	21 °C	37 °C
0	5.6 ± 0.01 ^AB^	5.6 ± 0.01	5.6 ± 0 ^AC^
1	5.6 ± 0 ^Aca^	5.6 ± 0 ^b^	5.6 ± 0 ^Ab^
3	5.7 ± 0.01 ^B^	5.6 ± 0.01	5.6 ± 0 ^AB^
6	5.7 ^B^	5.7 ± 0.01	5.7 ± 0.01 ^B^
24	5.8 ± 0.02 ^Ca^	5.6 ± 0.02 ^b^	5.6 ± 0.01 ^Cb^

The superscripts “^a,b,c^” represent differences between each solution across different storage temperatures at the same time point, while “^A,B,C,D^” represent differences in each storage condition over 24 h.

**Table 6:** pH (mean ± SEM) stability of hydrogen peroxide associated with amikacin or ciprofloxacin, both diluted in saline or lactated Ringer’s solution.

	H_2_O_2_ + AMK + saline (pH units)		
Time (h)	5 °C	21 °C	37 °C
0	6.6 ± 0.01 ^A^	6.6 ± 0.02 ^AB^	6.6 ± 0.01 ^A^
1	6.8 ± 0 ^Ba^	6.6 ± 0 ^Ab^	6.7 ± 0 ^Bc^
3	6.8 ± 0 ^Ba^	6.7 ± 0.01 ^Cb^	6.9 ± 0 ^Cc^
6	6.8 ± 0.01 ^Ba^	6.7 ± 0.01 ^BCb^	6.8 ± 0.01 ^Ca^
24	6.9 ± 0 ^Ca^	6.9 ± 0.01 ^Da^	7.2 ± 0.02 ^Db^
	H_2_O_2_ + AMK + lactated Ringer’s solution (pH units)		
Time (h)	5 °C	21 °C	37 °C
0	6.7 ± 0.01 ^A^	6.78 ± 0.01 ^A^	6.6 ± 0.01 ^A^
1	6.8 ± 0.02 ^B^	6.8 ± 0.01 ^AB^	6.7 ± 0 ^B^
3	6.9 ± 0.01 ^B^	6.8 ± 0 ^B^	6.9 ± 0 ^C^
6	6.9 ± 0.01 ^B^	6.8 ± 0.01 ^B^	6.9 ± 0.01 ^C^
24	7.1 ^Cab^	7.1 ± 0.01 ^Ca^	7.1 ± 0.01 ^Db^
	H_2_O_2_ + CIPRO + saline (pH units)		
Time (h)	5 °C	21 °C	37 °C
0	3.3 ± 0 ^A^	3.3 ± 0 ^ABC^	3.3 ± 0 ^A^
1	3.5 ± 0.01 ^Ba^	3.3 ± 0 ^Bb^	3.3 ± 0 ^Bc^
3	3.4 ± 0.01 ^Ba^	3.3 ± 0.01 ^Cb^	3.3 ± 0.01 ^ACb^
6	3.4 ± 0.01 ^BCa^	3.3 ± 0.01 ^BCb^	3.3 ± 0 ^Cb^
24	3.4 ± 0.01 ^Ca^	3.4 ± 0.01 ^Ab^	3.2 ± 0.1 ^BCab^
	H_2_O_2_ + CIPRO + lactated Ringer’s solution (pH units)		
Time (h)	5 °C	21 °C	37 °C
0	4.2 ± 0.01 ^A^	4.1 ± 0.01 ^A^	4.2 ± 0.01 ^A^
1	4.2 ± 0.01 ^BCa^	4.1 ± 0.01 ^ABb^	4.1 ± 0.01 ^Bc^
3	4.3 ± 0.01 ^Ca^	4.1 ± 0.01 ^Ab^	4.1 ± 0.01 ^ABc^
6	4.3 ± 0.01 ^Ca^	4.1 ± 0.01 ^Bb^	4.1 ± 0.01 ^Bb^
24	4.3 ^BDa^	4.2 ^ABb^	4.1 ± 0.01 ^ABb^

The superscripts “^a,b,c^” represent differences between each solution across different storage temperatures at the same time point, while “^A,B,C,D^” represent differences in each storage condition over 24 h.

**Table 7:** pH (mean ± SEM) stability of Tris-EDTA associated with amikacin, ceftiofur sodium, or ciprofloxacin diluted in saline or lactated Ringer’s solution.

	Tris-EDTA + AMK + saline (pH units)		
Time (h)	5 °C	21 °C	37 °C
0	7.1 ± 0.03 ^A^	7.1 ± 0.03 ^AB^	7.1 ± 0.03 ^A^
1	7.2 ± 0 ^Aa^	7.1 ± 0.01 ^Ab^	7.3 ± 0 ^Bc^
3	7.2 ± 0.01 ^Aa^	7.1 ± 0.01 ^Bb^	7.2 ± 0 ^Aab^
6	7.2 ± 0.01 ^Aa^	7.1 ± 0.01 ^Bb^	7.2 ± 0.01 ^Aa^
24	7.3 ± 0 ^Ba^	7.3 ± 0 ^Ca^	7.4 ± 0 ^Cb^
	Tris-EDTA + AMK + lactated Ringer’s solution (pH units)		
Time (h)	5 °C	21 °C	37 °C
0	7 ± 0.01 ^A^	7 ± 0.01 ^A^	7.0 ± 0.02 ^A^
1	7.2 ± 0 ^Ba^	7.1 ± 0.01 ^Ab^	7.3 ± 0 ^Bc^
3	7.2 ± 0.01 ^Ca^	7.1 ± 0.01 ^Bb^	7.2 ± 0.01 ^ACab^
6	7.2 ± 0.01 ^BCDa^	7.1 ± 0.01 ^Bb^	7.2 ± 0 ^Cab^
24	7.2 ± 0.01 ^Da^	7.3 ± 0.01 ^Cb^	7.4 ± 0 ^Dc^
	Tris-EDTA + CEFT + saline (pH units)		
Time (h)	5 °C	21 °C	37 °C
0	8.2 ^AB^	8.2 ± 0 ^A^	8.2 ± 0 ^A^
1	8.2 ± 0.01 ^ABa^	8.1 ± 0 ^Bb^	8.1 ± 0.01 ^Bc^
3	8.2 ± 0.01 ^ABa^	8.1 ± 0.01 ^BCb^	7.9 ± 0.01 ^BCc^
6	8.2 ± 0 ^Aa^	8.1 ± 0.01 ^BCb^	7.9 ± 0.01 ^Cc^
24	8.1 ± 0 ^Ba^	8.1 ± 0 ^Cb^	7.5 ± 0.01 ^Dc^
	Tris-EDTA + CEFT + lactated Ringer’s solution (pH units)		
Time (h)	5 °C	21 °C	37 °C
0	8.1 ± 0 ^A^	8.1 ± 0 ^A^	8.1 ± 0 ^A^
1	8.2 ± 0.01 ^ABa^	8.1 ± 0.01 ^ABb^	7.9 ± 0 ^Bc^
3	8.2 ± 0 ^BCa^	8.1 ± 0.01 ^ABCb^	7.9 ± 0.01 ^Cc^
6	8.2 ± 0.01 ^Ca^	8.1 ± 0 ^Bb^	7.8 ± 0.01 ^Cc^
24	8.2 ± 0.01 ^BCa^	8.1 ± 0 ^Cb^	7.5 ± 0 ^Dc^
	Tris- EDTA + CIPRO + saline (pH units)		
Time (h)	5 °C	21 °C	37 °C
0	7.40 ± 0 ^A^	7.4 ± 0 ^A^	7.4 ± 0 ^A^
1	7.1 ± 0.01 ^Ba^	7.1 ± 0.01 ^Bb^	6.9 ± 0.01 ^Bc^
3	7.2 ± 0.01 ^Ca^	7.1± 0 ^BCb^	6.9 ± 0.01 ^Cc^
6	7.2 ± 0.01 ^BCa^	7.1 ± 0.01 ^BCb^	6.9 ± 0.01 ^Cc^
24	7.3 ± 0.01 ^BCa^	7.1 ± 0.01 ^Cb^	6.9 ± 0.01 ^BCc^
	Tris-EDTA + CIPRO + lactated Ringer’s solution (pH units)		
Time (h)	5 °C	21 °C	37 °C
0	7.4 ± 0.01 ^A^	7.4 ± 0.01 ^A^	7.4 ± 0.01 ^A^
1	7.2 ± 0.02 ^Ba^	7.2 ± 0.01 ^Ba^	7.0 ± 0.01 ^BCb^
3	7.2 ± 0.01 ^Ba^	7.2 ± 0.01 ^BCb^	7.0 ± 0.01 ^Dc^
6	7.2 ± 0.01 ^Ba^	7.2 ± 0.01 ^Cb^	7.0 ± 0.01 ^Bc^
24	7.3 ± 0 ^Ca^	7.1 ^Db^	7.1 ± 0.01 ^CDc^

The superscripts “^a,b,c^” represent differences between each solution across different storage temperature over the same timepoint, while “^A,B,C,D^” represent differences in each storage condition over 24 h.

## Data Availability

The original contributions presented in this study are included in the article. Further inquiries can be directed to the corresponding author.

## Supplementary Materials

The following supporting information can be downloaded at: https://www.mdpi.com/article/10.3390/ani16030382/s1, Table S1. Antibiotics and the manufacturers from whom they were purchased; Table S2. Influence of storage temperature on pH (mean ± SEM) stability of antibiotic solutions over 24 h.
