# Supplementary material for: The Apparent pH Stability of Antibiotic Preparations Used for Uterine Infusions in Mares
Source: Animals (Basel). 2026 Jan 26;16(3):382. doi: 10.3390/ani16030382 (PMC12896860; doi:10.3390/ani16030382)
Supplement: Supplementary file 1 [file animals-16-00382-s001.zip › animals-3558261-supplementary.pdf]

Supplementary Materials

Table S1. Antibiotics and manufacturers from whom they were purchased.

Antibiotic	Manufacturer
Amikacin	Avet Pharma
Gentamicin	Vet One
Ceftiofur	Zoetis
Ciprofloxacin	Rood&Riddle Veterinary Pharmacy
Ampicillin	Rood&Riddle Veterinary Pharmacy
Penicillin G potassium	WG critical care
Penicillin G procaíne	Vet One
Ticarcillin clavulanate	Rood&Riddle Veterinary Pharmacy

Table S2. Influence of storage temperature on pH (mean ± SEM) stability of antibiotic solutions over 24 hours.

	AMK 1 g + saline (pH units)		
Time (h)	5°C	21°C	37°C
0	6.72 ^{AB}	6.71 ^{A}	6.7 ^{A}
1	6.85 ± 0.02 ^{Bab}	6.77 ± 0.01 ^{Ba}	6.82 ^{Bb}
3	6.96 ± 0.02 ^{Ca}	6.86 ± 0.01 ^{Cb}	6.75 ± 0.08 ^{ABCab}
6	7.09 ± 0.02 ^{D}	7.08 ± 0.01 ^{D}	7.13 ± 0.02 ^{C}
24	7.09 ^{D}	7.08 ^{D}	7.09 ^{C}
	AMK 1 g + lactated Ringer's (pH units)		
Time (h)	5°C	21°C	37°C
0	6.68 ± 0.01 ^{A}	6.7 ^{A}	6.7 ^{A}
1	6.87 ± 0.02 ^{Ba}	6.73 ± 0.02 ^{Ab}	6.58 ± 0.02 ^{Ac}
3	6.91 ± 0.01 ^{BCa}	6.78 ± 0.02 ^{Ab}	6.82 ^{Bb}
6	7.07 ± 0.03 ^{C}	7.05 ± 0.01 ^{BC}	7.05 ± 0.01 ^{C}
24	7.08 ± 0.02 ^{C}	7.08 ± 0.01 ^{C}	7.07 ^{C}
	AMK 2 g + saline (pH units)		
Time (h)	5°C	21°C	37°C
0	6.64 ± 0.01 ^{A}	6.64 ± 0.01 ^{A}	6.64 ± 0.01 ^{A}
1	6.69 ^{Ba}	6.57 ± 0.02 ^{Ab}	6.65 ± 0.03 ^{ABab}
3	6.75 ± 0.01 ^{BC}	6.76 ± 0.01 ^{B}	6.73 ± 0.01 ^{BC}
6	6.79 ± 0.01 ^{C}	6.79 ± 0.01 ^{B}	6.83 ± 0.01 ^{BCD}
24	6.81 ± 0.02 ^{C}	6.80 ± 0.02 ^{B}	6.88 ± 0.01 ^{D}
	AMK 2 g + lactated Ringer's (pH units)		
Time (h)	5°C	21°C	37°C
0	6.73 ± 0.01 ^{A}	6.72 ± 0.01 ^{A}	6.73 ± 0.01 ^{A}
1	6.80 ± 0.02 ^{Ba}	6.71 ± 0.01 ^{Ab}	6.75 ± 0.01 ^{Aa}
3	6.85 ^{BCa}	6.87 ± 0.01 ^{BCa}	6.81 ^{Ab}
6	6.83 ± 0.02 ^{ABC}	6.84 ± 0.01 ^{AC}	6.86 ± 0.02 ^{AC}
24	6.93 ± 0.01 ^{Cab}	6.89 ± 0.01 ^{BCa}	6.94 ^{BCb}
	AMP 1 g + saline (pH units)		
Time (h)	5°C	21°C	37°C
0	9.35 ± 0.01 ^{a}	9.39 ^{b}	9.4 ^{b}
1	9.12 ± 0.03 ^{a}	9 ^{a}	8.72 ± 0.02 ^{b}
3	8.97 ± 0.02 ^{a}	8.84 ^{b}	8.48 ± 0.01 ^{c}
6	8.98 ± 0.01 ^{a}	8.76 ± 0.01 ^{b}	8.38 ± 0.03 ^{c}

24	8.87 ± 0.01 ^{a}	8.54 ± 0.01 ^{b}	8.05 ± 0.03 ^{c}
AMP 1 g + lactated Ringer's (pH units)			
Time (h)	5°C	21°C	37°C
0	9.37 ± 0.02 ^{A}	9.37 ± 0.02 ^{A}	9.37 ± 0.02 ^{A}
1	9.14 ± 0.01 ^{Ba}	8.91 ± 0.01 ^{Bb}	8.67 ± 0.02 ^{Bc}
3	8.98 ± 0.01 ^{Ca}	8.87 ± 0.01 ^{Bb}	8.43 ± 0.01 ^{Bc}
6	9 ± 0.01 ^{Ca}	8.71 ± 0.01 ^{Cb}	8.32 ± 0.02 ^{Cc}
24	8.89 ± 0.01 ^{Da}	8.45 ± 0.01 ^{Db}	8.03 ± 0.01 ^{Dc}
AMP 2 g + saline (pH units)			
Time (h)	5°C	21°C	37°C
0	9.54 ± 0.01 ^{A}	9.54 ± 0.01 ^{A}	9.54 ± 0.01 ^{A}
1	9.30 ± 0.02 ^{Ba}	9.11 ± 0.01 ^{Bb}	8.80 ± 0.02 ^{Bc}
3	9.23 ± 0.02 ^{Ba}	8.91 ^{Cb}	8.63 ± 0.01 ^{Cc}
6	9.16 ± 0.01 ^{Ba}	8.77 ± 0.01 ^{Db}	8.45 ± 0.01 ^{Dc}
24	8.49 ± 0.01 ^{Ca}	8.51 ± 0.01 ^{Ea}	8.16 ± 0.01 ^{Eb}
AMP 2 g + lactated Ringer's (pH units)			
Time (h)	5°C	21°C	37°C
0	9.54 ^{A}	9.54 ^{A}	9.53 ± 0.01 ^{A}
1	9.35 ^{Ba}	9.03 ^{Bb}	8.69 ± 0.01 ^{Bc}
3	9.17 ± 0.02 ^{Ca}	8.91 ^{Cb}	8.57 ± 0.01 ^{Cc}
6	9.14 ± 0.01 ^{Ca}	8.72 ± 0.01 ^{Db}	8.32 ± 0.01 ^{Dc}
24	8.49 ± 0.01 ^{Da}	8.47 ± 0.01 ^{Ea}	8.15 ± 0.01 ^{Eb}
CEFT + saline (pH units)			
Time (h)	5°C	21°C	37°C
0	6.54 ± 0.01 ^{A}	6.54 ± 0.01 ^{A}	6.54 ± 0.01 ^{A}
1	6.58 ± 0.01 ^{Aa}	6.57 ± 0.01 ^{Aab}	6.54 ± 0.01 ^{Ab}
3	6.61 ^{ABa}	6.54 ± 0.02 ^{ABab}	6.44 ± 0.02 ^{ABb}
6	6.58 ± 0.02 ^{ABa}	6.58 ± 0.02 ^{ABa}	6.47 ^{Bb}
24	6.64 ± 0.01 ^{Ba}	6.52 ± 0.01 ^{Bb}	6.23 ± 0.01 ^{Cc}
CEFT + lactated Ringer's (pH units)			
Time (h)	5°C	21°C	37°C
0	6.60 ± 0.01 ^{A}	6.60 ± 0.01 ^{A}	6.60 ± 0.01 ^{A}
1	6.65 ± 0.02 ^{B}	6.63 ± 0.02 ^{A}	6.59 ± 0.01 ^{A}
3	6.59 ± 0.01 ^{ABa}	6.62 ± 0.01 ^{Aa}	6.54 ± 0.01 ^{Bb}
6	6.64 ± 0.02 ^{ABa}	6.61 ± 0.01 ^{Aa}	6.50 ^{Bb}
24	6.66 ± 0.02 ^{ABa}	6.42 ± 0.01 ^{Bb}	6.24 ± 0.01 ^{Cc}
CIPRO + saline (pH units)			
Time (h)	5°C	21°C	37°C
0	4.34 ± 0.01 ^{A}	4.35 ^{A}	4.34 ± 0.01 ^{A}
1	4.41 ± 0.01 ^{ABa}	4.36 ^{Ab}	4.17 ^{Bc}
3	4.40 ^{Ba}	4.21 ^{ABCb}	4.17 ^{Bb}
6	4.41 ^{ABa}	4.18 ^{Bb}	4.18 ^{Bb}
24	4.14 ^{Ca}	4.10 ^{Cb}	4.10 ^{Cb}
CIPRO + lactated Ringer's (pH units)			
Time (h)	5°C	21°C	37°C
0	5.02 ± 0.02 ^{A}	5.02 ± 0.02	5.02 ± 0.02
1	5.08 ± 0.01 ^{ABa}	4.99 ± 0.02 ^{ab}	4.97 ± 0.01 ^{b}
3	5.12 ^{Ba}	5 ± 0.01 ^{b}	4.96 ± 0.01 ^{c}
6	5.15 ^{Ca}	5.01 ± 0.01 ^{b}	4.98 ^{b}
24	5.09 ± 0.06 ^{ABC}	5 ± 0.01	4.98
GEN 1 g + saline (pH units)			

Time (h)	5°C	21°C	37°C
0	6.50 ± 0.01 ^{A}	6.50 ± 0.01 ^{A}	6.50 ± 0.01 ^{A}
1	6.74 ± 0.03 ^{ABC}	6.68 ± 0.01 ^{B}	6.66 ± 0.02 ^{B}
3	6.77 ± 0.03 ^{A}	6.75 ± 0.01 ^{C}	6.76 ± 0.02 ^{B}
6	6.78 ± 0.01 ^{Ba}	6.92 ± 0.01 ^{Db}	6.93 ^{Cb}
24	7. 01 ± 0.02 ^{Ca}	6.98 ± 0.02 ^{Da}	7.16 ± 0.01 ^{Db}
		GEN 1 g + lactated Ringer's (pH units)	
Time (h)	5°C	21°C	37°C
0	6.47 ± 0.01 ^{A}	6.47 ± 0.01 ^{A}	6.47 ± 0.01 ^{A}
1	6.70 ± 0.01 ^{Bab}	6.76 ± 0.02 ^{Ba}	6.64 ± 0.03 ^{Ab}
3	6.69 ± 0.01 ^{Ba}	6.74 ± 0.02 ^{Bab}	6.86 ± 0.03 ^{Bb}
6	6.85 ± 0.01 ^{Ca}	6.87 ^{BCa}	6.9 ^{Bb}
24	6.99 ± 0.01 ^{Da}	6.89 ± 0.01 ^{Cb}	7.15 ± 0.02 ^{Cc}
		GEN 2 g + saline (pH units)	
Time (h)	5°C	21°C	37°C
0	6.40 ^{A}	6.40 ^{A}	6.40 ^{A}
1	6.50 ± 0.01 ^{Aa}	6.51 ± 0.01 ^{Ba}	6.48 ± 0.01 ^{Bb}
3	6.57 ^{Ba}	6.56 ± 0.01 ^{Cab}	6.53 ^{Bb}
6	6.58 ± 0.01 ^{B}	6.57 ± 0.01 ^{BC}	6.54 ± 0.01 ^{BC}
24	6.59 ± 0.01 ^{B}	6.61 ± 0.01 ^{C}	6.56 ± 0.02 ^{C}
		GEN 2 g + lactated Ringer's (pH units)	
Time (h)	5°C	21°C	37°C
0	6.40 ± 0.01 ^{A}	6.40 ± 0.01 ^{A}	6.39 ± 0.01 ^{A}
1	6.51 ^{Ba}	6.44 ± 0.01 ^{Bb}	6.47 ± 0.01 ^{Bc}
3	6.51 ± 0.01 ^{B}	6.56 ± 0.02 ^{C}	6.52 ^{B}
6	6.60 ± 0.01 ^{Ca}	6.58 ± 0.01 ^{Ca}	6.73 ^{Cb}
24	6.61 ^{Ca}	6.59 ± 0.01 ^{Ca}	6.70 ^{Cb}
		KPEN + saline (pH units)	
Time (h)	5°C	21°C	37°C
0	7.34 ± 0.02 ^{AB}	7.34 ± 0.02 ^{A}	7.34 ± 0.02 ^{A}
1	7.23 ± 0.02 ^{Aa}	7.30 ± 0.02 ^{Aa}	7.02 ± 0.01 ^{Bb}
3	7.20 ± 0.01 ^{ABa}	7.07 ^{Bb}	6.64 ± 0.02 ^{Cc}
6	7.28 ± 0.04 ^{ABa}	7.01 ± 0.01 ^{Cb}	6.47 ± 0.01 ^{Dc}
24	7.15 ± 0.02 ^{Ba}	6.66 ± 0.01 ^{Db}	5.27 ± 0.01 ^{Ec}
		KPEN + lactated Ringer's (pH units)	
Time (h)	5°C	21°C	37°C
0	7.30 ^{ABa}	7.31 ^{Aa}	7.30 ^{Ab}
1	7.27 ± 0.01 ^{Aa}	7.31 ^{Ab}	7.02 ± 0.02 ^{Bc}
3	7.30 ± 0.02 ^{Aa}	7.04 ± 0.02 ^{Bb}	6.76 ± 0.01 ^{Cc}
6	7.21 ± 0.02 ^{Ba}	7.04 ± 0.01 ^{Bb}	6.54 ± 0.01 ^{Dc}
24	7.2 ± 0.02 ^{Ba}	6.69 ± 0.01 ^{Cb}	5.46 ± 0.03 ^{Ec}
		PPG+ saline (pH units)	
Time (h)	5°C	21°C	37°C
0	6.38 ± 0.01	6.39 ± 0.01 ^{AB}	6.40 ^{A}
1	6.39 ± 0.01	6.40 ± 0.01 ^{A}	6.30 ± 0.03 ^{AB}
3	6.41 ± 0.01	6.42 ± 0.01 ^{B}	6.30 ± 0.01 ^{B}
6	6.40 ± 0.01 ^{a}	6.40 ± 0.01 ^{Aa}	6.28 ± 0.01 ^{Bb}
24	6.40 ± 0.01 ^{a}	6.37 ± 0.01 ^{Ca}	6.27 ± 0.01 ^{Bb}
		PPG + lactated Ringer's (pH units)	
Time (h)	5°C	21°C	37°C
0	6.38 ± 0.02 ^{AB}	6.38 ± 0.02 ^{AB}	6.38 ± 0.02 ^{AB}

1	6.43 ± 0.02 [Ca]	6.36 ± 0.01 [Ab]	6.36 ± 0.01 [ABCb]
3	6.36 ± 0.01 [BCa]	6.42 ± 0.01 [Bab]	6.42 ± 0.01 [ACc]
6	6.41 [Aa]	6.41 [ABa]	6.41 [BDb]
24	6.42 ± 0.01 [Aa]	6.42 ± 0.01 [ABa]	6.41 ± 0.01 [CDb]
	TIC-CLAV + saline (pH units)		
Time (h)	5°C	21°C	37°C
0	6.22 ± 0.01 [A]	6.22 ± 0.01 [AB]	6.22 ± 0.01 [A]
1	6.20 [Aa]	6.23 [Ab]	6.20 [Aa]
3	6.21 [Aa]	6.22 [ABab]	6.20 [Ab]
6	6.37 ± 0.01 [Ba]	6.20 [ABb]	6.10 [Bc]
24	6.39 ± 0.01 [Ba]	6.20 [Bb]	6.09 [Bc]
	TIC-CLAV + lactated Ringer's (pH units)		
Time (h)	5°C	21°C	37°C
0	6.10 [A]	6.09 [A]	6.10 [A]
1	6.10 ± 0.01 [A]	6.09 ± 0.01 [A]	6.08 ± 0.01 [A]
3	6.11 ± 0.01 [A]	6.09 [A]	6.08 [AB]
6	6.31 [Ba]	6.16 [Bb]	6 ± 0.01 [BCc]
24	6.40 [Ca]	6.26 ± 0.01 [Cb]	5.96 ± 0.01 [Cc]

The superscripts "a, b, c" indicate differences between antibiotic solutions at different storage temperatures within the same timepoint, while "A, B, C, D" indicate differences within the same storage temperature across different timepoints over 24 hours.Experiment 2. Influence of antibiotic' concentration on solutions' stability.
